# Validation of the forced swim test in *Drosophila*, and its use to demonstrate psilocybin has long-lasting antidepressant-like effects in flies

**DOI:** 10.1038/s41598-022-14165-2

**Published:** 2022-06-15

**Authors:** M. Hibicke, C. D. Nichols

**Affiliations:** Department of Pharmacology and Experimental Therapeutics, LSU Health Sciences Center, New Orleans, LA USA

**Keywords:** Neuroscience, Pharmacology

## Abstract

Psilocybin has been shown to be a powerful, long-lasting antidepressant in human clinical trials and in rodent models. Although rodents have commonly been used to model psychiatric disorders, *Drosophila* have neurotransmitter systems similar to mammals and many comparable brain structures involved in similar behaviors. The forced swim test (FST), which has been used extensively to evaluate compounds for antidepressant efficacy, has recently been adapted for *Drosophila*. The fly FST has potential to be a cost-effective, high-throughput assay for evaluating potential antidepressants. For this study we pharmacologically validated the fly FST using methamphetamine, DL-α-methyltyrosine, and the antidepressant citalopram. While methamphetamine and DL-α-methyltyrosine altered overall locomotor activity in the *Drosophila* Activity Monitor System (DAMS), they had no significant impact on measures of immobility in the FST. Conversely, chronic citalopram decreased measures of immobility in the FST in both sexes without increasing DAMS activity. We used the validated FST to evaluate the antidepressant-like effects of high (3.5 mM) and low (0.03 mM) doses of psilocybin. Both doses of psilocybin significantly reduced measures of immobility in male flies, but not females. 0.03 mM had an effect size comparable to chronic citalopram, and 3.5 mM had an effect size approximately twice that of chronic citalopram.

## Introduction

Rats are commonly used for behavioral models of human disease states, and have been traditionally preferred to model psychiatric disorders^1^. Although powerful in behavioral paradigms, rat models have not been developed for extensive genetic remodeling in the way that the classic genetic model, *Drosophila melanogaster*, has been developed^2^. *Drosophila* neurotransmitter systems are remarkably similar to mammalian systems^3^, they have many comparable brain structures involved in similar behaviors^4^, and many genes known to influence mammalian behavior are present and performing the same function in *Drosophila*^5^. The conservation of neurotransmitter system across phylum manifests as similar responses to drugs^6^, but *Drosophila* has not yet been fully utilized for high-throughput behavioral screening of potential pharmacotherapies.

The forced swim test (FST) is a behavioral despair paradigm frequently used in rats to measure coping strategies in response to inescapable adversity, wherein a passive coping strategy (immobility) is interpreted as depressive-like, and reductions immobility as evidence of antidepressant-like effect^7,8^. The FST can be used to screen for antidepressant action of drugs given to otherwise healthy rats^8,9^, for passive coping strategies in animal models of depression^10,11^, or for antidepressant-like effects of drugs given to animals used for the study of depression^11–13^. Although lack of neurocircuit-specific modulations of FST behaviors reduces translational value, due to behavioral sexual dimorphism, the FST is the most reliable measure of depressive-like behavior in both male and female rats^9,14^. Further, the assay has shown strong face and predictive validity in that risk factors for human depression have repeatedly been shown to increase immobility in the FST^11,15^, and antidepressant therapies efficacious in reducing human depression also reduce immobility in the FST^8,11,15^. As the FST measures active and passive coping strategies, it is potentially confounded by non-specific sedative or stimulant effects of drugs that affect overall activity levels^7^. For example, psychostimulant amphetamine increases active behaviors in the rodent FST along with overall locomotor activity, and antipsychotic sedative chlorpromazine increases immobility in the FST while decreasing overall locomotor activity. However, antidepressant drugs selectively increase active behaviors in the FST without increasing overall locomotor activity^7,8^. Thus, any investigation into the effects of putative antidepressants requires evaluation of overall locomotor activity to exclude confounding by nonspecific stimulant effects.

Recently, the forced swim test (FST) has been adapted for use in *Drosophila*^16,17^. There is evidence that *Drosophila* exposed to specific stressors display increased immobility in the FST without overall reductions in locomotor activity^16,18^, suggesting face validity for the fly FST as a measure of behavioral despair, passive coping strategies that are interpreted as depressive-like behavior. However, the fly adaptation of the FST had yet to be pharmacologically validated for predictive validity. The fly FST, if validated, may become a powerful tool for elucidating the pharmacological mechanisms responsible for the effects of antidepressant drugs, and for describing the neurocircuitry involved in changing passive to active coping strategies. Additionally, it has potential to become an extremely cost-effective, high-throughput screening assay for behavioral evaluation of antidepressant-like effects of potential pharmacotherapies, such as psychedelics like psilocybin.

Psychedelic research is currently undergoing a renaissance. Representative compounds have been shown to be powerful, persistent antidepressants in humans^19–22^, have antidepressant-like effects^13^ and to reduce inflammation in rodents^23–25^, and to increase neuroplasticity in larval *Drosophila* and rodent neuronal cell culture^26^.

In this study we pharmacologically evaluate the predictive validity of the fly FST using the psychostimulant methamphetamine (METH), the functional sedative DL-α-methyltyrosine (αMT), the established selective serotonin reuptake inhibitor (SSRI) antidepressant citalopram (CIT), as well as the psychedelic prodrug psilocybin (PSI), the 5-HT_1A_ receptor antagonist WAY 100635 (WAY), and the 5-HT_2_ receptor antagonist ketanserin (KET). METH increases synaptic activity by reversing the transport direction of the sodium-dependent dopamine, norepinephrine, and serotonin transporters, and increases *Drosophila* locomotor activity^27^. αMT inhibits the enzyme tyrosine hydroxylase, resulting in catecholamine depletion, and decreases *Drosophila* locomotor activity^28^. CIT selectively inhibits the sodium-dependent serotonin receptor (SERT), and has been shown to bind to SERT in *Drosophila* (dSERT)^29^. Psilocybin is converted by alkaline phosphatase to its active metabolite psilocin. Psilocin binds with high affinity to 5-HT_2A_ receptors, and with lower affinity to other serotonergic receptors, including 5-HT_1A_. Ketanserin and WAY 100635 have both been shown to be bioactive in *Drosophila*^30^, and were used to explore the pharmacological mechanism by which psilocybin affects locomotor activity.

## Results

### Forced swim behaviors and overall locomotor activity vary by sex, strain, and drug response

**Figure 1:**
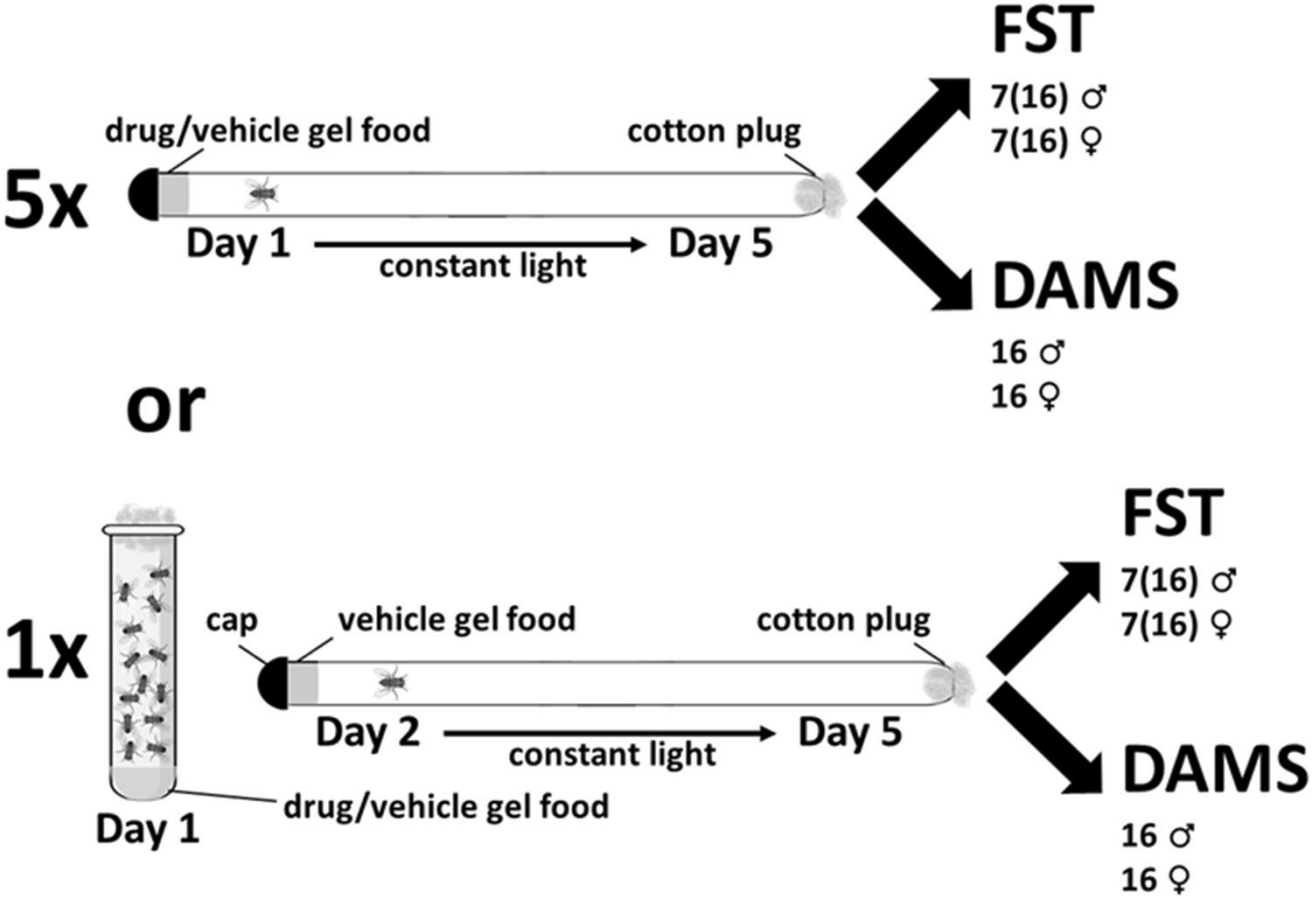
Experimental design for pulsed (1×) and chronic (5×) dosing. One-day old male and female flies were placed individually into transparent tubes plugged with a loose wad of cotton on one end, and with transparent food medium and a plastic cap on the other end. Food medium was either vehicle, METH (5.0 mM), αMT (3.0 mM), CIT (0.3, 1.0, or 2.5 mM), PSI (0.03 mM, 3.5 mM), KET (1.0 mM), or WAY (1.0 mM). 1× indicates that flies were fed for 24 h on treatment food under group conditions, then transferred into a tube containing vehicle medium (described above). 5× indicates that flies were fed for 5 consecutive days on treatment food in tube as described above. Flies were placed into the DAMS in groups of *n* = 16 for each sex in each treatment group, and kept at constant light conditions for at least 5 days while being monitored. All other flies were kept under constant light conditions for 5 days, then tested in the FST so that *n* = 7 for each sex in each treatment group, but the individual data points in the FST represent the mean behaviors of 16 flies. A total of 896 flies were used to generate *n* = 7 for males (448) and females (448) for each treatment group (112/group).

The *Drosophila* Activity Monitor System (DAMS) was used to measure locomotor activity over time, and the fly forced swim test (FST) was used to measure immobility behaviors in 6-day old males and females of two laboratory standard wild type *Canton-Special* (*CS*) flies of distinct lineages. Flies were fed on vehicle medium for 5 days or on a pulsed dose of psilocybin, where the flies fed on psilocybin (0.03 mM) in vehicle medium for 1 day (1×), then vehicle medium for 4 days (Fig. 1).

**Figure 2:**
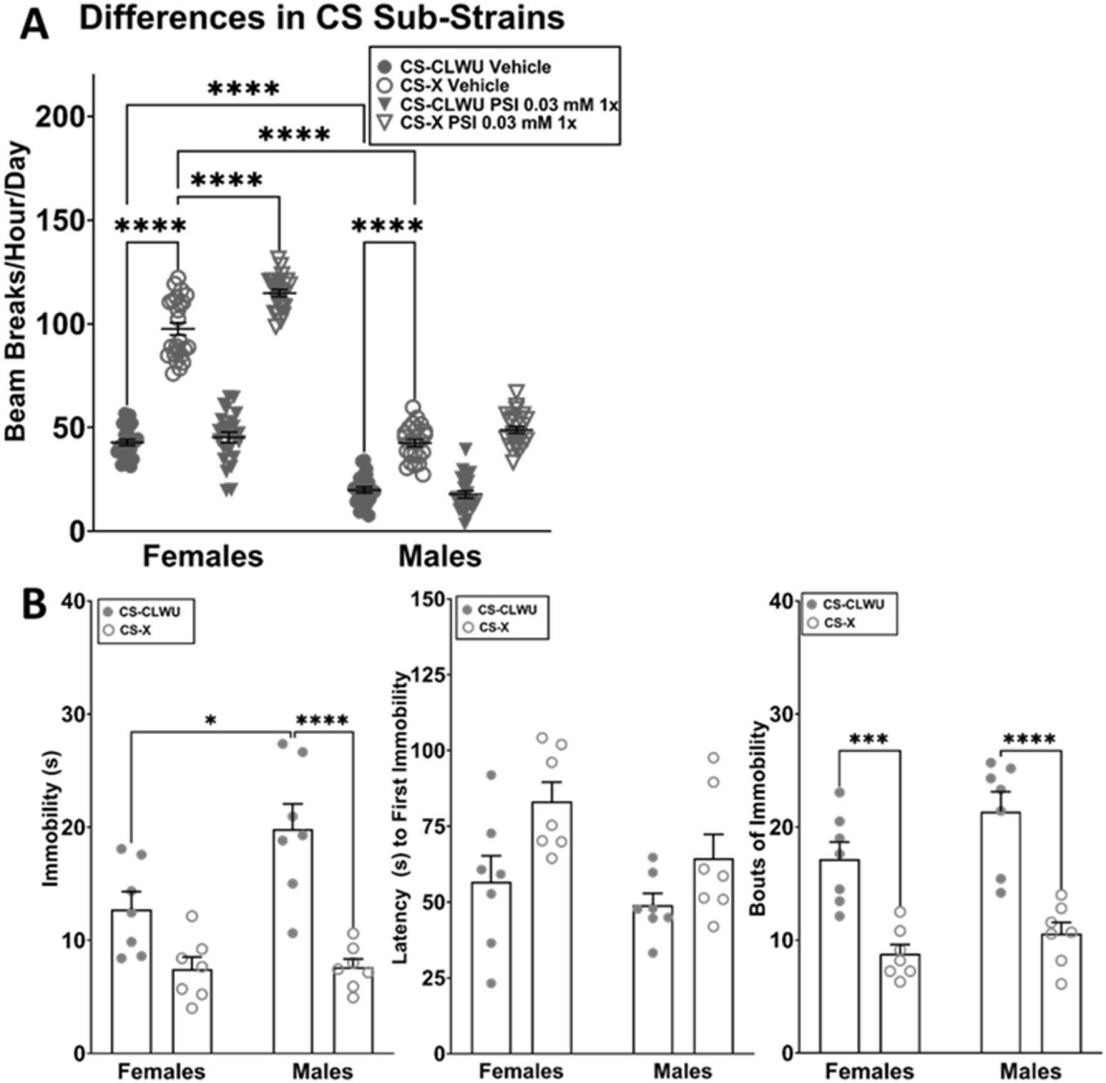
Differences in *CS* sub-strains in the DAMS and FST. The locomotor activity and forced swim responses of 6-day-old flies from two distinct *CS* lineages (*CS*^*CLWU*^ and *CS*^*X*^) were compared under control and PSI (0.03 mM) 5× conditions, 5 days after administration. Data were evaluated using 2-way ANOVA; *p < 0.05, ***p < 0.001, ****p < 0.0001. (**A**) When fed vehicle medium, *CS*^*X*^ flies of both sexes were more active than *CS*^*CLWU*^, and males of both strains were less active than females. PSI increased locomotor activity in female *CS*^*X*^ flies. No other relevant differences were observed. (**B**) *CS*^*CLWU*^ males were significantly more immobile than *CS*^*CLWU*^ females and *CS*^*X*^ males. *CS*^*CLWU*^ flies had significantly more bouts of immobility than *CS*^*X*^. No other relevant differences were observed.

In the DAMS, a two-way ANOVA for the independent variables sex (male, female) and treatment (vehicle, PSI 0.03 mM) found a significant interaction between sex and treatment (*p* < 0.0001), as well as significant variation due to sex (*p* < 0.0001) and treatment (*p* < 0.0001). *CS*^*X*^ females (*p* < 0.0001) and males (*p* < 0.0001) fed vehicle medium were significantly more active than same-sex *CS*^*CLWU*^ counterparts (Fig. 2A). Both *CS*^*X*^ (*p* < 0.0001) and *CS*^*CLWU*^ (*p* < 0.0001) males were significantly less active than same-strain females. PSI (0.03 mM) increased locomotor activity in *CS*^*X*^ females (*p* < 0.0001), but not in *CS*^*X*^ males or *CS*^*CLWU*^ flies. No other relevant differences were observed.

In the FST, a two-way ANOVA for the independent variables sex (male, female) and strain (*CS*^*X*^, *CS*^*CLWU*^) found a significant interaction between sex and strain (*p* = 0.0306), as well as significant variation due to sex (*p* = 0.0249) and strain (*p* < 0.0001). *CS*^*CLWU*^ males were significantly more immobile than *CS*^*CLWU*^ females (*p* = 0.0115) and *CS*^*X*^ males (*p* < 0.0001), but no differences were observed between male and female *CS*^*X*^ flies (Fig. 2B). *CS*^*X*^ females (*p* = 0.0007) and males (*p* < 0.0001) had significantly fewer bouts of immobility than their same-sex *CS*^*CLWU*^ counterparts (Fig. 2B). No other relevant differences were observed.

As *CS*^*CLWU*^ flies given 0.03 PSI (1×) were more immobile in the FST and did not increase their overall locomotor activity in the DAMS, that strain was selected for additional experimentation. Additionally, as male *CS*^*CLWU*^ flies were significantly (and consistently) more immobile than their female counterparts, sexes were evaluated independently in subsequent FST analyses.

### Immobility in the forced swim is independent of overall locomotor activity, and decreased by citalopram

The predictive validity of the fly FST was evaluated by comparing the immobility behaviors of male and female *CS*^*CLWU*^ flies fed for 5 days (5×) on vehicle or drug in vehicle medium before being tested for immobility behaviors in the FST. FST behaviors were compared with overall locomotor activity on the fifth day in the DAMS of flies fed on vehicle medium or drug in vehicle medium (Fig. 1). Drugs used were METH (5.0 mM), αMT (3.0 mM), and CIT (0.3 mM, 1.0 mM, 2.5 mM).

**Figure 3:**
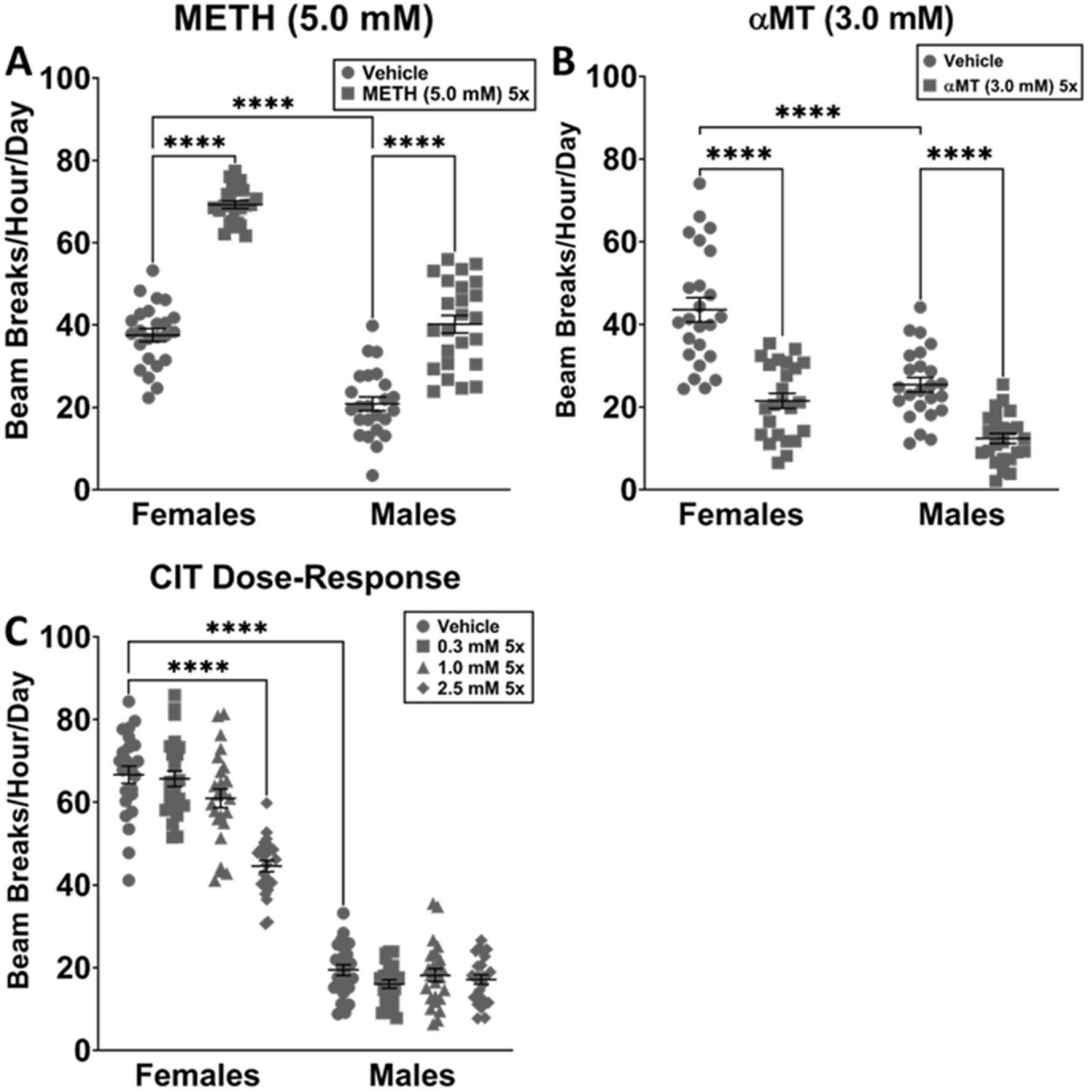
Locomotor activity as measured by DAMS for METH, αMT and CIT. The changes in overall locomotor activity caused by dopaminergic modulators METH (5.0 mM) 5× and αMT (3.0 mM) 5× were compared with those caused by SSRI CIT (0.3, 1.0, and 2.5 mM) 5×. Data were evaluated with 2-way ANOVA; ****p < 0.0001. (**A**) *n* = 16 for each group. Control males were significantly less active than control females. METH significantly increased locomotor activity in both males and females. (**B**) *n* = 16 for each group. Control males were significantly less active than control females. αMT significantly decreased locomotor activity in both males and females. (**C**) *n* = 16 for each group but females CIT (0.3 mM), in which *n* = 15. Control males were significantly less active than control females. CIT (2.5 mM) significantly decreased locomotor activity in females, but no other differences were observed among groups.

Two-way ANOVA for the independent variables sex (male, female) and treatment (vehicle, METH) found a significant interaction between sex and treatment (*p* = 0.0003), as well as significant variation introduced by both sex (*p* < 0.0001) and treatment (*p* < 0.0001). Males fed vehicle medium were significantly less active than females fed vehicle medium (*p* < 0.0001). METH significantly increased locomotor activity in both males (*p* < 0.0001) and females (*p* < 0.0001) (Fig. 3A). No other relevant differences were observed.

Two-way ANOVA for the independent variables sex (male, female) and treatment (vehicle, αMT) found a significant interaction between sex and treatment (*p* = 0.0268), as well as significant variation due to sex (*p* < 0.0001) and treatment (*p* < 0.0001). Males fed vehicle medium were significantly less active than females fed vehicle medium (*p* < 0.0001). αMT significantly decreased locomotor activity in both males (*p* < 0.0001) and females (*p* < 0.0001) (Fig. 3B). No other relevant differences were observed.

Two-way ANOVA for the independent variables sex (male, female) and treatment (vehicle, 0.3, 1.0, and 2.5 mM CIT) found significant interaction between sex and treatment (*p* < 0.0001), as well as significant variation introduced by both sex (*p* < 0.0001) and treatment (*p* < 0.0001). Males fed vehicle medium were significantly less active than females fed vehicle medium (*p* < 0.0001). 2.5 mM CIT significantly decreased locomotor activity in females (*p* < 0.0001), but no other differences were observed among groups. 1.0 mM was chosen for use in the FST (Fig. 2C).

**Figure 4:**
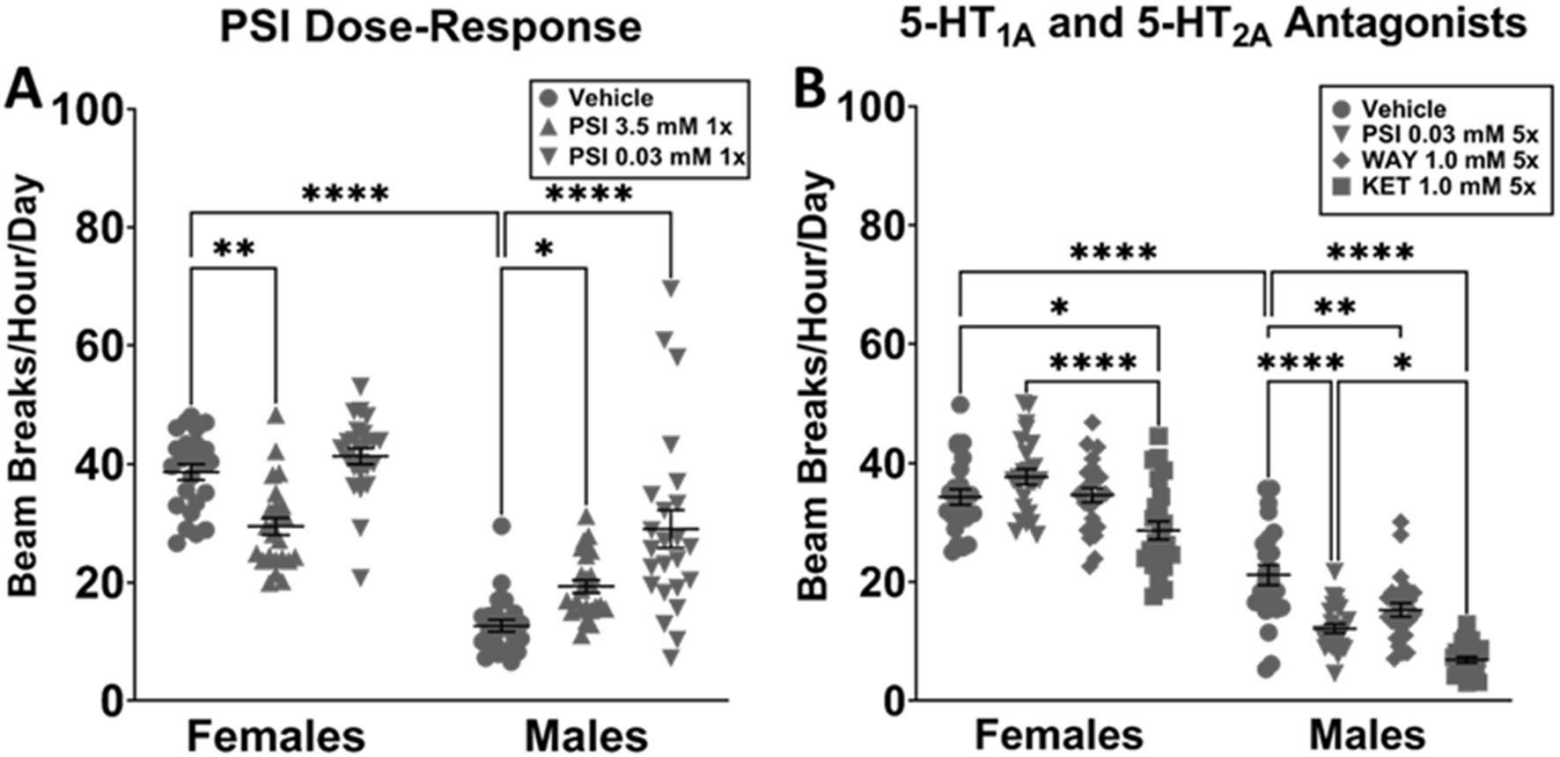
Use of METH, αMT, and CIT to establish predictive validity of FST. The changes in metrics of immobility in the forced swim caused by dopaminergic modulators METH (5.0 mM) 5× and αMT (3.0 mM) 5× were compared with those caused by SSRI CIT (3.0 mM) 5×. Sexes were evaluated independently with one-way ANOVA and Holm-Sidak post-hoc; *n* = 7 for each group, and each of 7 data points is the mean of 16 flies. *p < 0.05, **p < 0.01, ***p < 0.001, ****p < 0.0001. CIT significantly decreased male immobility vs vehicle, but no other relevant differences were observed. CIT increased latency to first immobility in females and males vs vehicle, but no other relevant differences were observed. CIT significantly decreased bouts of immobility in males vs vehicle, but no other relevant differences were observed.

As males were consistently significantly less active at baseline in the DAMS (Figs.2a, 3a–c), FST behaviors of the sexes were evaluated independently using one-way ANOVA. CIT significantly decreased immobility in males (*p* = 0.0137), increased latency to first immobility in females (*p* = 0.0227) and males (*p* = 0.0051), and decreased bouts of immobility in males (*p* = 0.0285). No significant differences in any outcome measure were observed for either METH or αMT compared to vehicle (Fig. 4).

### Overall locomotor response to psilocybin varies by sex, dose, and dosing strategy

**Figure 5:**
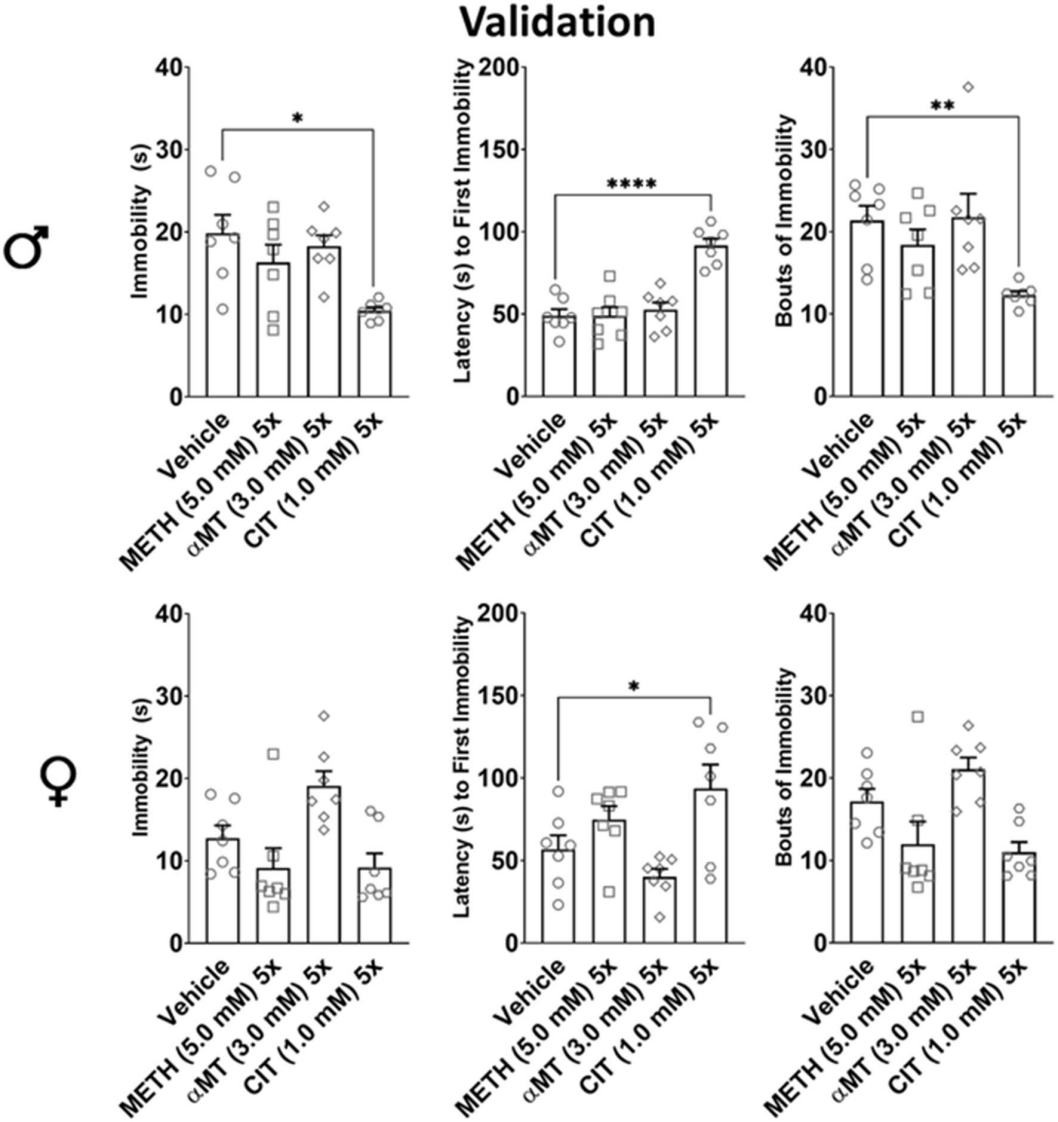
Locomotor activity as measured by DAMS for PSI, WAY, and KET. The changes in overall locomotor activity caused by low (0.03 mM) 1× and high (3.5 mM) 1× dose PSI were assessed, as were those caused by PSI (0.03 mM) 5×, WAY (1.0 mM) 5×, and KET (1.0 mM) 5×. Data were evaluated with 2-way ANOVA; *p < 0.05, **p < 0.01, ****p < 0.0001. (**A**) Female PSI (3.5 mM) *n* = 15; all other groups *n* = 16. Control males were significantly less active than control females. 3.5 mM PSI significantly decreased locomotor activity in females. 3.5 mM and 0.03 mM PSI significantly increased locomotor activity in males. No other relevant differences were observed. (**B**) Female vehicle *n* = 15; all other groups *n* = 16. Control males were significantly less active than control females. KET significantly decreased locomotor activity vs vehicle in females vs vehicle and PSI (0.03 mM). PSI, WAY, and KET significantly decreased locomotor activity in males. Both sexes given KET were significantly less active than males given PSI. No other relevant differences were observed.

To evaluate the effects of PSI on overall locomotor activity in the DAMS, PSI (0.03 mM, 3.5 mM) was pulse administered (1×) to be consistent with dosing strategies for antidepressant effect in clinical trials^21,22^ and in research with rats^13^. Two-way ANOVA for the independent variables sex (male, female) and treatment (vehicle, PSI 3.5 mM, PSI 0.03 mM) found a significant interaction between sex and treatment (*p* < 0.0001) (Fig. 5A), as well as significant variation due to sex (*p* < 0.0001) and treatment (*p* < 0.0001). Control males were significantly less active than females (*p* < 0.0001). PSI decreased locomotor activity in females at 3.5 mM (*p* = 0.0011), but increased locomotor activity in males at 3.5 mM (*p* = 0.0237) and 0.003 mM (*p* < 0.0001).

### Chronic and pulsed low-dose psilocybin affect overall locomotor activity differently

To assess whether PSI’s DAMS effects were due to actions at the 5-HT_1A_ or 5-HT_2A_ serotonin receptors, flies were fed for 5 days (5×) on PSI (0.03 mM), WAY (1.0 mM), KET (1.0 mM), or on vehicle medium. Two-way ANOVA for the independent variables sex (male, female) and treatment (vehicle, PSI 0.03 mM, KET 1.0 mM, WAY 1.0 mM) found a significant interaction between sex and treatment (*p* < 0.0001), as well as significant variation due to sex (*p* < 0.0001) and treatment (*p* < 0.0001) (Fig. 5B). Control males were significantly less active than females (*p* < 0.0001). KET significantly decreased locomotor activity in females (*p* = 0.0102) and males (*p* < 0.0001). WAY significantly decreased locomotor activity in males (*p* = 0.0075) but not females. Females (*p* < 0.0001) and males (*p* = 0.0167) given PSI were significantly more active than their same-sex counterparts given KET. No other differences were observed (Fig. 5B).

### Psilocybin’s effects on immobility in the forced swim were comparable to those of citalopram

**Figure 6:**
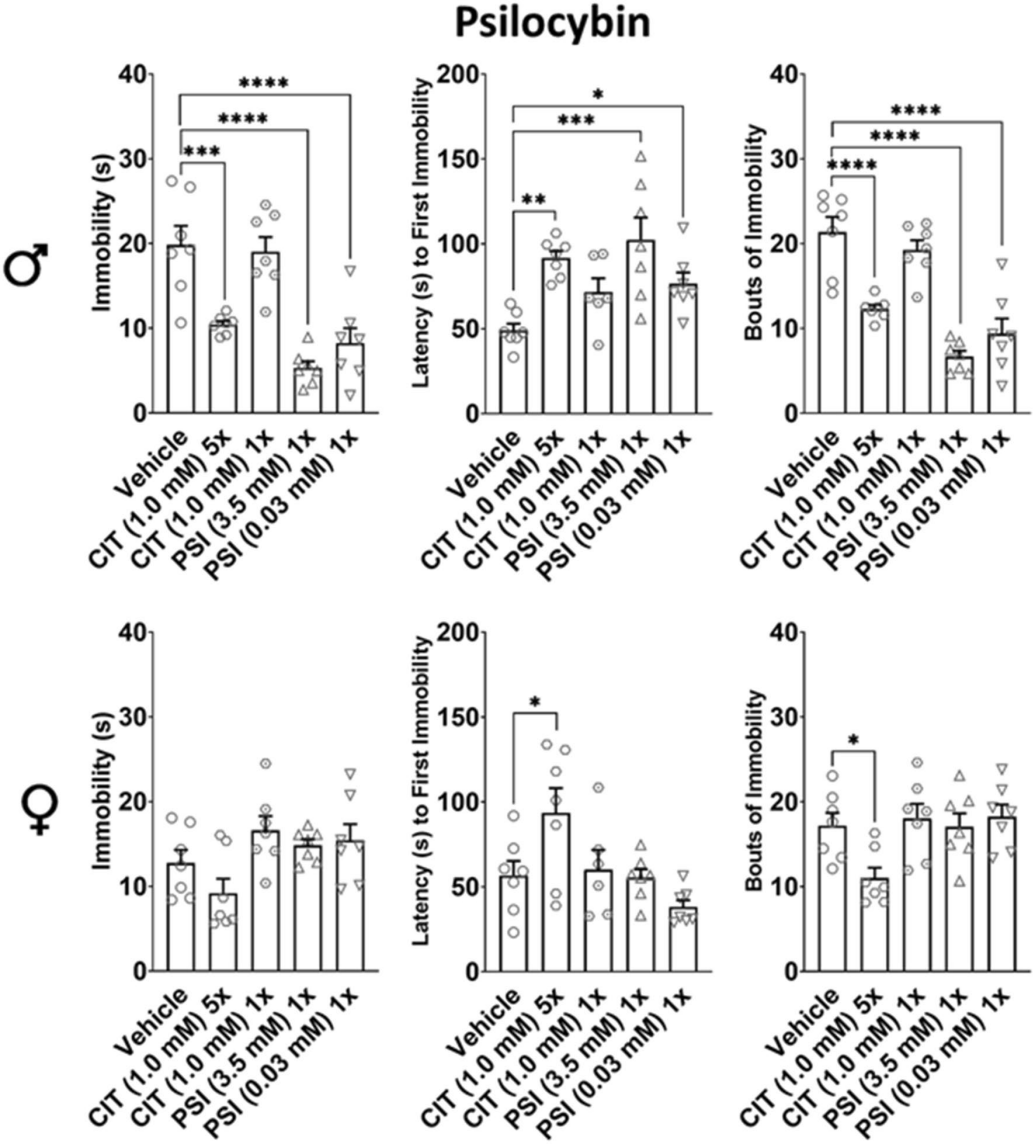
Comparison of PSI with CIT in the FST. The changes in metrics of immobility in the forced swim caused by SSRI CIT (3.0 mM) 5× and 1× were compared with those caused by PSI (3.5 mM) 1× and (0.03 mM) 1×. Sexes were evaluated independently with one-way ANOVA and Holm-Sidak post-hoc; *n* = 7 for each group, and each of 7 data points is the mean of 16 flies. *p < 0.05, **p < 0.01, ****p < 0.0001. Males given CIT 5×, and both high and low PSI were significantly less immobile than vehicle, but no differences were observed in males given CIT 1× or in any females. Males, and females given CIT 5×, and males given both high PSI and low PSI displayed greater latency to first immobility vs vehicle. Males and females given CIT 5×, and males given both high PSI and low PSI displayed significantly fewer bouts of immobility than vehicle.

CIT 5 × significantly reduced immobility (*p* = 0.0004), latency to first immobility (*p* = 0.0018), and bouts of immobility (*p* < 0.0001) in males, and significantly reduced latency to first immobility (*p* = 0.0362) and bouts of immobility (*p* = 0.0267) in females. PSI significantly reduced immobility at 3.5 mM (*p* < 0.0001) and 0.03 mM (*p* < 0.0001), latency to first immobility at 3.5 mM (*p* = 0.0002) and 0.03 mM (*p* = 0.0382), and bouts of immobility at 3.5 mM (*p* < 0.0001) and 0.03 mM (*p* < 0.0001) in males. No other differences were observed (Fig. 6).

## Discussion

METH (Fig. 3A) and α MT (Fig. 3A) significantly altered locomotor activity in both male and female flies, but largely did not affect FST behaviors (Fig. 4). Comparatively, CIT (1.0 mM) 5× did not affect locomotor activity in either male or female flies (Fig. 3C), but significantly decreased male immobility, latency to immobility in both sexes, and male bouts of immobility (Fig. 4). Thus, activity in the FST is decoupled from overall locomotor activity, and is selectively modulated by an SSRI antidepressant drug (Fig. 4). Prior work by Araujo, et al. has provided face and some construct validity^18^, and the results of our study indicate predictive validity for Neckameyer and Nieto-Romero’s Drosophila adaptation of the FST^16^.

Both high and low doses of PSI 1× profoundly reduced male immobility behaviors in the FST (Fig. 6), but also increased overall locomotor activity in the DAMS. In the typical usage of the FST, antidepressant drugs selectively increase activity in the FST but not overall locomotor activity^8^. However, in typical usage of the FST, the drug being investigated has been administered within 24 h of testing. In this experiment, PSI was administered for a single day, and then the flies were administered vehicle medium for 4 days prior to testing. As *CS*^*CLWU*^ males appear to be exceptionally immobile when compared to females and males of another strain (Fig. 2), increased locomotor activity in both the DAMS and the FST may indicate a gain of function and subsequent normalization of behavior. Supporting this hypothesis, PSI (0.03 mM) 5×, as well as WAY and KET 5× reduced male DAMS activity (Fig. 5B). Chronic antagonism of 5-HT_1A_ receptors by WAY and of 5-HT_2_ receptors by KET resulted in similar behavioral outcomes as chronic PSI (an agonist at both receptors, which is apparently contradictory. Paradoxically, both agonists and antagonists have been known to downregulate these receptors^31–33^. Thus, the observed reductions in DAMS activity may be consequences of receptor internalization (Fig. 5B).

While the FST has traditionally been an excellent predictor of antidepressant-like activity of investigational drugs, behaviors in the assay are not neurocircuit-specific, and there has been recent controversy regarding its use^34^. However, *Drosophila* has been well-developed as a model organism for genetic modulation with low translational value for biomedical purposes. Concurrent use of genetic modulations with behavioral assays validated for *Drosophila* provides greater translational value and strengthens the integrity of *Drosophila* as a model organism for biomedical research. So while the *Drosophila* adaptation of the FST will likely never be used as an independent measure of drug action to determine neural circuitries in mammalian systems underlying the FST, as a complement to investigations of targeted gene expression, the fly FST can help elucidate which genetic modulations are behaviorally relevant and inform on fundamental shared mechanisms of neuronal modulation leading to specific changes in behaviors.

The FST’s use in *Drosophila* is promising, but some potential confounding factors exist. For example, significant strain and sex differences in baseline locomotor activity (Fig. 2A) and drug response in the FST (Fig. 2B). While some of these differences may be attributable to non-neurological differences, such as females having greater fat deposition and thus greater buoyancy, the extent to which sexually dichotomous neurological differences contribute to behavioral differences has yet to be established. As *Drosophila* research traditionally uses female flies exclusively in an experiment only when investigating reproductive-related functions, our understanding of sexually dichotomous drug responses is limited. Additionally, the FST uses three different metrics—total immobility, latency to first immobility, and bouts of immobility. While established antidepressant CIT 5× affects each metric in male *Drosophila* (Figs. 4, 6), they are unaffected in females. How these data are best interpreted is unclear. Further, pulsed dosing of experimental antidepressant PSI 1× has effects similar to, but more pronounced than, CIT 5× (Fig. 6), suggesting that PSI enacts persistent increases in serotonergic signaling. Future directions should include further elucidation of sex differences, as well as neurocircuit-specific investigations using targeted genetic modulations of 5-HT receptors.

## Conclusions

We have validated the* Drosophila* FST for use with CNS-active drugs, in that we have demonstrated that activity in the FST is decoupled from overall locomotor activity as measured by the DAMS, and is sensitive to modulation by SSRI antidepressant citalopram. Chronic exposure to citalopram is necessary to reduce immobility, bouts of immobility, and to increase latency to first immobility. This is similar to SSRI effects in humans, where chronic dosing is necessary to produce an antidepressant effect. Further, the effects of citalopram on measures of immobility are conserved with rodents in the FST. Significantly, we found that psilocybin has an antidepressant-like effect in flies, with at least equal effect size as the antidepressant citalopram at both high and low feeding concentrations. That these effects were long-lasting after a single 24 h exposure, similar to psilocybin’s antidepressant efficacy measured in human clinical trials where effects persist 6 months or more after a single treatment^21,22^, indicates potential translational relevance for the fly as a model to study psychedelic drug neuropharmacology. Moreover, the fly adaptation of the FST may be suitable for detecting assay-specific behavioral effects of antidepressants and/or antidepressant-like drugs in general, and the assay can be used in complement with neurocircuit-specific studies to elucidate which neurocircuit modulations are behaviorally relevant.

## Methods

### Experimental design

Groups of one-day old, non-entrained flies were fed for 5 days (5×) on a medium of 1% agarose, 10% sucrose containing METH (5.0 mM), αMT (3.0 mM), CIT (0.3, 1.0, or 2.5 mM), PSI (0.03 mM), WAY (1.0 mM), or KET (1.0 mM) in vehicle medium, or for 1 day (1×) on CIT (1.0 mM) or PSI (0.03 or 3.5 mM), then vehicle medium until testing. Control flies were fed vehicle medium gel consisting of 10% sucrose and 1% agarose (Fig. 1). Flies were observed eating the food under all conditions. Five days after first treatment, 16 male and 16 female flies from each treatment group were evaluated for locomotor activity using the *Drosophila* Activity Monitor System (DAMS, TriKinetics Inc, Waltham, MA USA), so that *n* = 16 for each group, excluding individuals that died within the array prior to data collection. For the FST analysis, 5 days after first treatment, seven groups of 16 male and 16 female flies from each treatment group were evaluated in the FST, and the means of each group of 16 flies tested in the FST were used as single data points to represent that population, so that *n* = 7 for each treatment group (Fig. 1). A total of 896 flies were used to generate *n* = 7 for males (448) and females (448) for each treatment group (112/group).

### Drugs and reagents

Citalopram was chosen as a model antidepressant because it has been shown to bind to the dSERT^29^, and has previously been used to elicit significant behavioral changes in *Drosophila*^35^. Methamphetamine is a psychostimulant shown to increase locomotor activity in *Drosophila*^27^ and in rats^36^. αMT is a tyrosine hydroxylase inhibitor previously shown to reduce locomotor activity in *Drosophila*^28^. Psilocybin is an experimental psychedelic shown to have powerful antidepressant effects in humans^19–22^ that can be modeled in animals^13^. Dosage for CIT was determined by guidance from the literature^29^ and dose–response measuring locomotor activity. Dosage for METH, αMT, PSI, KET, and WAY were determined by guidance from the literature^13,28,30,35^ and confirmed by previous use in our laboratory^27,30^.

### Flies

*Canton-special* (*CS*) flies were used to evaluate the validity of the paradigm within a wild type approximation. Flies were kept under constant light conditions at room temperature to avoid potential circadian-related confounds. Freshly eclosed adult flies were lightly anesthetized with carbon dioxide to be separated by sex into larger storage vials in preparation for placement according to treatment group and assay. As different sub-strains of *CS* flies have been shown to display locomotor activity differences^37^, two sub-strains of *CS* (*CS*^*X*^ and *CS*^*CLWU*^) were compared for general locomotor activity and immobility behaviors in the forced swim test, and the sub-strain with greater immobility was selected. *CS*^*CLWU*^ flies were originally supplied by Professor Chunlai Wu of the Neuroscience Center of Excellence at the School of Medicine, LSU New Orleans. *CS*^*X*^ flies were originally supplied by Dr. Wendi Neckamayer (St. Louis University). Both strains had been cultured in our laboratory for many years prior to this study.

### *Drosophila* Activity Monitor System

One-day old (used in the ‘5×’ 5 day feeding paradigm) or two-day old (used in the ‘1×’ 1 day feeding paradigm) adult flies were briefly anesthetized and placed individually into 5 mm diameter transparent glass or plastic tubes plugged with a loose wad of cotton on one end and capped with transparent food medium containing METH (5.0 mM), α MT (3.0 mM), CIT (0.3, 1.0, or 2.5 mM) on the other end (Fig. 1). The tubes were then placed into the *Drosophila* Activity Monitor System (DAMS) unit. Each unit contains slots for 32 tubes, with an infrared beam array that monitors beam breaks within each tube using DAMSystem 2 software (TriKinetics Inc, Waltham, MA USA). Locomotor activity for Day 5 is reported as average beam breaks per hour per day.

#### Methamphetamine

One-day old (5×), non-entrained flies were briefly anesthetized and placed individually into glass DAMS tubes capped with METH (5.0 mM) in vehicle or vehicle food medium, then evaluated in the DAMS for 5 days.

#### DL-α-methyltyrosine

One-day old (5×), non-entrained flies were briefly anesthetized and placed individually into glass DAMS tubes (previously described) capped with αMT (3.0 mM) in vehicle or vehicle food medium, then evaluated in the DAMS for five days.

#### Citalopram dose–response

One-day old (5×), non-entrained flies were briefly anesthetized and placed individually into glass DAMS tubes capped with CIT (0.3, 1.0, or 2.5 mM) in vehicle or vehicle food medium, then evaluated in the DAMS for 5 days. The highest CIT dosage that did not affect locomotor activity at Day 5 was chosen for use in the FST.

#### Psilocybin dose–response

One-day old (1×), non-entrained flies were sorted by sex and fed on PSI (0.03 or 3.5 mM) in vehicle medium for 1 day, then briefly anesthetized and placed individually into glass DAMS tubes capped with vehicle medium, and evaluated in the DAMS until Day 5. Control flies were placed into DAMS tubes with vehicle food on Day 1.

#### 5-HT_1A_ and 5-HT_2A_ antagonists

One-day old (5×), non-entrained flies were briefly anesthetized and placed individually into glass DAMS tubes capped with PSI (0.03 mM), WAY (1.0 mM), KET (1.0 mM) in vehicle or vehicle medium, and evaluated in the DAMS for 5 days.

### Forced swim test

One-day old (5×) or 2-day old (1×), non-entrained flies were briefly anesthetized and placed individually into plastic DAMS tubes. The tubes were then placed cotton up into the rack of a 200 mL pipet tip box and left under constant light conditions until Day 5. FST behaviors were evaluated placing one fly per well in 4-well chamber slides containing 1.5 mL 0.08% room temperature SDS in each well^16,18^. An overhead camera was used to record FST behaviors during the first 5 min in the well for later analysis for latency to first immobility, time spent immobile, and number of bouts of immobility using EthoVision XT 8.5 (Noldus Technology, Leesburg, VA, USA). To verify that the flies were capable of mobility, each fly was gently removed with a spatula and flicked onto a paper napkin after the 5 min of video recording. Any flies unable to immediately walk away from their landing locations were assumed drowned and subsequently excluded from final analysis^16^.

#### Validation

Establishing predictive validity for the FST requires demonstrating that immobility behaviors in the FST are decoupled from overall locomotor activity and can be selectively modulated by an established antidepressant drug. We used psychostimulant METH (which increases overall locomotor activity in the DAMS), functional sedative αMT (which reduces overall locomotor activity in the DAMS), and established SSRI antidepressant CIT (at a dose of 1.0 mM that does not affect locomotor activity in the DAMS). One-day old (5×) flies were fed on vehicle or medium containing METH (5.0 mM), αMT (3.0 mM), CIT (1.0 mM) for 5 days, then tested in the FST.

#### Psilocybin

Using a pulsed dosing strategy, PSI has been shown to be a powerful antidepressant in humans^21,22^ and to profoundly reduce immobility in rats^13^, but SSRIs like CIT require chronic administration for antidepressant efficacy in humans. Thus, PSI (0.03 and 3.5 mM) 1× were compared with both CIT (1.0 mM) 1× and CIT (1.0 mM) 5× , with the expectation that CIT 1× flies would not be different from control flies.

### Statistical analysis

DAMS and *CS*^*CLWU*^ vs *CS*^*X*^ FST data were compared using two-way analysis of variance (ANOVA) with Holm-Sidak post hoc in Prism software (Graphpad, La Jolla, CA) for the independent variables of sex (male, female) and treatment (vehicle, CIT, METH, α MT, PSI 3.5 mM, PSI 0.03 mM). Other FST data were evaluated using one-way ANOVA, and the sexes were evaluated independently. For the DAMS, *n* = 16 for all groups, male and female, prior to exclusions. For the FST, *n* = 7 for all groups, where each data point represents the mean of 16 individuals. Data are expressed as mean ± standard error of the mean (SEM).

### Exclusions

During the CIT dose–response in the DAMS, one female in the 0.3 mM treatment group was dead by Day 5, so for that treatment group *n* = 15. Immediately following forced swim testing, each fly was scooped out of the well with a small spatula and gently flicked onto a paper towel. Any fly unable to immediately get up and walk away was excluded from analysis and did not contribute to the data or n values presented in this manuscript.

## Data Availability

The datasets used and/or analyzed during the current study available from the corresponding author on request.
